# Disrupted resting-state brain network properties in obesity: decreased global and putaminal cortico-striatal network efficiency

**DOI:** 10.1017/S0033291716002646

**Published:** 2016-11-02

**Authors:** K. Baek, L. S. Morris, P. Kundu, V. Voon

**Affiliations:** 1 Department of Psychiatry, University of Cambridge, Addenbrooke's Hospital, Cambridge CB2 0QQ, UK; 2 Behavioural and Clinical Neurosciences Institute, University of Cambridge, Cambridge CB2 0QQ, UK; 3 Departments of Radiology and Psychiatry, Icahn School of Medicine at Mount Sinai, New York City, NY 10029, USA; 4 Cambridgeshire and Peterborough NHS Foundation Trust, Cambridge CB21 5EF, UK; 5 NIHR Cambridge Biomedical Research Centre, Cambridge CB2 0QQ, UK

**Keywords:** Binge eating, brain networks, graph theory, obesity, resting-state functional magnetic resonance imaging

## Abstract

**Background:**

The efficient organization and communication of brain networks underlie cognitive processing and their disruption can lead to pathological behaviours. Few studies have focused on whole-brain networks in obesity and binge eating disorder (BED). Here we used multi-echo resting-state functional magnetic resonance imaging (rsfMRI) along with a data-driven graph theory approach to assess brain network characteristics in obesity and BED.

**Method:**

Multi-echo rsfMRI scans were collected from 40 obese subjects (including 20 BED patients) and 40 healthy controls and denoised using multi-echo independent component analysis (ME-ICA). We constructed a whole-brain functional connectivity matrix with normalized correlation coefficients between regional mean blood oxygenation level-dependent (BOLD) signals from 90 brain regions in the Automated Anatomical Labeling atlas. We computed global and regional network properties in the binarized connectivity matrices with an edge density of 5%–25%. We also verified our findings using a separate parcellation, the Harvard–Oxford atlas parcellated into 470 regions.

**Results:**

Obese subjects exhibited significantly reduced global and local network efficiency as well as decreased modularity compared with healthy controls, showing disruption in small-world and modular network structures. In regional metrics, the putamen, pallidum and thalamus exhibited significantly decreased nodal degree and efficiency in obese subjects. Obese subjects also showed decreased connectivity of cortico-striatal/cortico-thalamic networks associated with putaminal and cortical motor regions. These findings were significant with ME-ICA with limited group differences observed with conventional denoising or single-echo analysis.

**Conclusions:**

Using this data-driven analysis of multi-echo rsfMRI data, we found disruption in global network properties and motor cortico-striatal networks in obesity consistent with habit formation theories. Our findings highlight the role of network properties in pathological food misuse as possible biomarkers and therapeutic targets.

## Introduction

The resting-state brain network shows functional topological features such as small-world and modular organization, which enables efficient information processing and communication through the network (Achard *et al.* 2006; Achard & Bullmore, 2007; Bullmore & Sporns, 2009, 2012). Graph-theoretical analysis of resting-state functional magnetic resonance imaging (rsfMRI) data reveals the topological properties of whole-brain functional networks in a data-driven manner. While the application of graph-theoretic analysis to the brain networks is still relatively new, brain network properties in rsfMRI measurements have been found to be disrupted in various neuropsychiatric disorders such as Alzheimer's disease (Supekar *et al.* 2008; Yao *et al.* 2010), schizophrenia (Liu *et al.* 2008; van den Heuvel *et al.* 2010), major depression (Zhang *et al.* 2011) and attention-deficit/hyperactivity disorder (Wang *et al.* 2009).

Here, we aimed to examine alterations in brain network properties in individuals with obesity with and without binge eating disorder (BED). BED is a compulsive eating behaviour characterized by rapid food intake that has been hypothesized in preclinical models to have overlaps with disorders of addiction (Gearhardt *et al.* 2011; Avena *et al.* 2012; Smith & Robbins, 2013). Across both human and rodent studies of binge eating, the striatum and dopaminergic system has been implicated as a crucial mediator of problematic and compulsive eating behaviours. In rodent studies of binge eating, repeated access to sucrose is associated with increased dopamine signalling in the ventral striatum (Hajnal & Norgren, 2002; Rada *et al.* 2005) and reduced dopamine D_2_ receptor binding in the dorsolateral striatum (Bello *et al.* 2002). Rodents with a knockdown of dorsolateral striatum dopamine D_2_ receptor expression show compulsive food intake (Johnson & Kenny, 2010). In humans with BED, food stimuli elicit a similar enhancement of striatal dopamine release (Wang *et al.* 2011). Finally, reduced striatal D_2/3_ receptor availability has been demonstrated in humans with obesity (de Weijer *et al.* 2011), a feature that is common for both obesity with and without BED.

However, little is known about alterations in resting-state brain networks in obesity and pathological behaviours towards food. While there are no known graph-theoretic analyses of brain networks in obesity and BED, some evidence of aberrant functional organization comes from studies using independent component analysis of rsfMRI data. Obese individuals seem to show increased connectivity strength of the putamen (Garcia-Garcia *et al.* 2013) but this was in a relatively small sample (*n* = 16). Functional connectivity strength of the left orbitofrontal cortex and right putamen was positively associated with fasting insulin levels and negatively with insulin sensitivity across obese and lean individuals (Kullmann *et al.* 2012), thereby suggesting a role for putaminal network dynamics in the regulation of food intake. Disrupted network organization has been also implicated in disorders of addiction, which may be potential markers that may be expressed in obesity as well. For example, decreased small-world characteristic and/or reduced global efficiency was observed in heroin-dependent individuals (Liu *et al.* 2009; Jiang *et al.* 2013) and drug-dependent subjects (Wang *et al.* 2015). Heavy smokers also displayed decreased global efficiency and increased local efficiency in the network (Lin *et al.* 2015). Finally, pathological gamblers demonstrated regional alteration of network properties in the paracingulate gyrus and supplementary motor area (SMA) (Tschernegg *et al.* 2013).

In the current study, we examine global and regional network properties of the resting-state brain network in 40 obese subjects (including 20 obese BED patients) in comparison with 40 matched healthy controls in a data-driven approach using graph theory analysis and network-based statistics (NBS) (Zalesky *et al.* 2010). We hypothesize that subjects with obesity (or BED) will have disrupted topological properties in cortical–striatal networks with particular implications for the dorsolateral striatum or putamen. We also use a recently developed multi-echo fMRI acquisition and multi-echo independent component analysis (ME-ICA) which improves signal quality via removing non-blood oxygenation level-dependent (BOLD) noise (Kundu *et al.* 2013), since previous graph-theoretic studies of compulsive behaviours may be disadvantaged by limited sample sizes and low signal:noise ratio with conventional single-echo rsfMRI.

## Method

### Participants

A total of 40 obese subjects [body mass index (BMI) >30 kg/m^2^] and 40 age- and gender-matched healthy controls (BMI of 18.1–25.9 kg/m^2^) were recruited via community- and university-based advertisements in Cambridge (See Table 1 for demographic information). Of the 40 obese subjects, 20 were identified as BED patients using Research Diagnostic Criteria from the Diagnostic and Statistical Manual of Mental Disorders, version IV (American Psychiatric Association, 2000). In the data analysis, we first compared all 40 obese subjects *v*. healthy controls, and subsequently compared the two obese subgroups, i.e. 20 obese BED patients *v*. 20 obese subjects without BED. Other psychiatric disorders were screened with the Mini International Neuropsychiatric Interview (Sheehan *et al.* 1998). Participants were excluded if they had a current major depressive episode or another major psychiatric disorder including substance addiction, major medical illness, or were taking psychotropic medication. The National Adult Reading Test was used to assess intelligence quotient (IQ). Participants completed the Binge Eating Scale (BES; Gormally *et al.* 1982) and the Beck Depression Inventory (BDI; Beck & Beamesderfer, 1974). Participants were reimbursed for their time and written informed consent was obtained. The study was approved by the University of Cambridge Research Ethics Committee. The authors assert that all procedures contributing to this work comply with the ethical standards of the relevant national and institutional committees on human experimentation and with the Helsinki Declaration of 1975, as revised in 2008.

**Table 1.** Demographic and clinical characteristics

	Study group			
Characteristic	BED (*n* = 20)	Obesity without BED (*n* = 20)	Healthy controls (*n* = 40)	*p*
Male gender, *n* (%)	9 (45)	13 (65)	20 (50)	n.s.
Age, years	43.7 (9.6)	42.7 (11.1)	41.8 (11.7)	n.s.
Body mass index, kg/m^2^	33.0 (2.4)	33.4 (3.9)	22.5 (2.0)	<0.001
Binge Eating Scale	23.2 (7.8)	11.0 (9.6)	5.0 (4.5)	<0.001
IQ	115.7 (7.1)	115.1 (5.8)	120.5 (4.4)	0.015
Beck Depression Inventory	14.8 (7.2)	8.0 (8.0)	4.7 (5.1)	<0.001

Data are given as mean (standard deviation) unless otherwise indicated.

BED, Binge eating disorder; n.s., non-significant; IQ, intelligence quotient.

### Multi-echo rsfMRI

We acquired BOLD fMRI data during wakeful rest for 10 min in all participants. During the rsfMRI scan, participants were asked to fixate on the white cross on the black background shown on the screen. To enhance signal:noise ratio, we utilized a novel multi-echo planar imaging sequence and independent components analysis (ME-ICA) in which BOLD signal components were identified with linear echo time (TE) dependency in the rsfMRI signal (Kundu *et al.* 2012, 2013). Data were acquired with a Siemens 3T Tim Trio scanner using a 32-channel head coil at the Wolfson Brain Imaging Centre at the University of Cambridge. T1-weighted anatomical images were acquired using a magnetization prepared rapid gradient echo (MPRAGE) sequence [176 × 240 field of view (FOV); 1-mm in-plane resolution; inversion time, 1100 ms]. Functional images were acquired with a multi-echo planar imaging sequence with online reconstruction [repetition time, 2.47 s; flip angle, 78°; matrix size 64 × 64; in-plane resolution, 3.75 mm; FOV, 240 mm; 32 oblique slices, alternating slice acquisition slice thickness 4.0 mm with 10% gap; integrated parallel imaging techniques (iPAT) factor, 3; band width = 1698 Hz/pixel; TE = 12, 28, 44 and 60 ms].

Data preprocessing was conducted using ME-ICA (ME-ICA v2.5 beta10; http://afni.nimh.nih.gov) (Kundu *et al.* 2013). The anatomical image was first skull-stripped and then was non-linearly warped to the Montreal Neurological Institute (MNI) anatomical template using AFNI. Motion correction and anatomical–functional co-registration was conducted in the functional data of the shortest TE using AFNI. The functional data were normalized to the MNI template using the non-linear warping computed from the anatomical image. After preprocessing the dataset of each TE, multi-echo rsfMRI data were decomposed with FastICA into approximately independent components, and non-BOLD components were identified with TE-dependency. BOLD contrast is associated with a change in the transverse relaxation rate R*_2_ induced by a change in blood oxygenation, which is linearly dependent with TE. In contrast, non-BOLD signal intensity changes are independent of TE. *F* values for these TE-dependent and -independent factors were computed in a voxel-wise manner for each component, and were summarized into two pseudo *F* statistics; *κ* and *ρ*, respectively (Kundu *et al.* 2012). Then, BOLD components were identified as the components with higher *κ* and lower *ρ* using thresholds derived from rank orderings (*κ*-spectrum and *ρ*-spectrum). Non-BOLD components which had lower *κ* and higher *ρ* were removed. After preprocessing with ME-ICA, we applied a high-pass filter (>0.01 Hz) on the denoised rsfMRI data.

To assess the effectiveness of ME-ICA denoising, we also tested a conventional single-echo fMRI denoising method. In this single-echo fMRI analysis, the data underwent the same preprocessing but the non-BOLD components (which presumably include motor artifact) determined by ME-ICA were not excluded. Then, we regressed out six head movement parameters and their temporal derivatives (frame-wise motion) and applied a bandpass filter of 0.01–0.1 Hz range. The same graph theory analysis was conducted in this dataset processed with a conventional denoising method.

### Graph theory analysis

Graph-theoretical analysis reveals the topological properties of whole-brain networks in a data-driven manner. In this framework, the brain network is usually deconstructed into multiple brain regions and connections between them, which are nodes and edges in the graph, respectively. Using the Automated Anatomical Labeling (AAL) atlas (Tzourio-Mazoyer *et al.* 2002), we divided the whole brain except the cerebellum into 90 (45 for each hemisphere) cortical and subcortical regions to define the nodes of the network. Separately, we performed the same network construction using the Harvard–Oxford Atlas with even-sized parcellations of 470 regions (H-O470) used in a previous study (Patel & Bullmore, 2015) to confirm the findings in graph theory metrics. We estimated Pearson's correlation *r*_*i,j*_ between the regional mean rsfMRI signals from the brain regions (nodes) *i* and *j*. Then, we normalized *r*_*i,j*_ using Fisher's *r*-to-*z* transform, resulting in *z*_*i,j*_, a 90 × 90 functional connectivity matrix for each subject for AAL and a 470 × 470 matrix for the H-O470 atlas.

In most graph-theoretical studies, global thresholding is used to construct a binarized network (Bullmore & Sporns, 2009; Rubinov & Sporns, 2010) in order to control the number of edges in the network across subjects. If the element *z*_*i,j*_ of the functional connectivity matrix is greater than a threshold *τ*, the corresponding element of the binarized network matrix, *a*_*i,j*_, is set to 1, otherwise it is set to 0. A varying level of threshold *τ* creates a graph with a different edge density *s*, which is the ratio of the number of edges existing in the network to the maximum number of possible edges, *n*(*n* − 1)/2 for a graph consisting of *n* nodes.

In order to balance an appropriate level of sparseness in the networks for all subjects, we determined the range of edge density (5% ⩽ *s* ⩽ 25%) in which the network of the healthy control group holds the small-world property according to the following criteria: (1) the average number of edges (degree) over all nodes 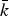 in the binarized network was larger than log(*N*) (Watts & Strogatz, 1998; Jiang *et al.* 2013); and (2) the normalized local efficiency of the network for each healthy control was higher than 1 (see below for the definition). We calculated each graph theory parameter along the edge density range of 5% ⩽ *s* ⩽ 25% with an increment of 1% and then averaged it into a summarized scalar over the above range.

We calculated the following graph theory metrics in the binarized networks using the Brain Connectivity Toolbox (http://www.brain-connectivity-toolbox.net) (Rubinov & Sporns, 2010) with MATLAB software (http://www.mathworks.com): (1) global network properties: global efficiency, local efficiency, modularity, normalized global efficiency, normalized local efficiency; (2) regional (nodal) network properties: nodal degree, nodal efficiency and nodal betweenness centrality.

#### Global network properties

Efficiency is a measure of parallel information transfer in the network which is more biologically relevant for the brain functional network. Efficiency of information transfer between nodes *i* and *j* can be defined as the inverse of the shortest path length *L*_*i,j*_, the number of edges in the shortest path between nodes *i* and *j*. Efficiency has a value between 0 (no path is available between nodes *i* and *j*) and 1 (nodes *i* and *j* are directly connected with an edge). Global efficiency (*E*_*glob*_) of the network G is defined as the average value of efficiency for all pairs of nodes in the network as defined as the following: 
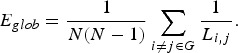


Local efficiency of node *i* can be defined using the same efficiency metric in the subgraph *G*_*i*_ which is consisting of the neighbouring nodes of the node *i* as the following: 
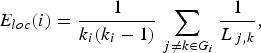
 where *k*_*i*_ is the degree of node *i*, the number of edges linked with the node *i* (i.e. the number of neighbouring nodes). Since the subgraph *G*_*i*_ does not include the node *i*, local efficiency can be considered as the a measure of fault tolerance indicating how efficient the communication is without node *i*. Local efficiency of the entire network (*E*_*loc*_) was calculated as the average of local efficiency across all nodes in the network.

The resting-state brain network has been reported to be one of small-world networks which lies somewhere between a random and a regular network and has high efficiency in both global and local scales (Latora & Marchiori, 2001; Achard *et al.* 2006; Achard & Bullmore, 2007). Compared with a random network, a small-world network retains local clustering organization (i.e. higher local efficiency than a random network) but also has short path lengths via a few shortcut connections (i.e. global efficiency comparable with a random network). To confirm small-world properties of the network, we calculated the normalized efficiency, the ratio between the efficiency of the original network and the efficiency of a randomly rewired network [*E*(*orig.*)/*E*(*random*)]. As a small-world network has higher *E*_*loc*_ than a random network, normalized local efficiency, *E*_*loc*_(*orig.*)/*E*_*loc*_(*random*), is expected to be higher than 1 (normalized *E*_*loc*_ > 1). In contrast, a small-world network has *E*_*glob*_ similar to a random network, normalized global efficiency, *E*_*glob*_(*orig.*)/*E*_*glob*_(*random*), should be near 1 (normalized *E*_*glob*_ ≈ 1). To calculate normalized local and global efficiency, we generated 100 random control networks for each network by randomly rewiring edges in the network when preserving the degree of each node. Then, we estimated normalized *E*_*glob*_ and *E*_*loc*_ as the ratio of real *E*_*glob*_ and *E*_*loc*_ to average *E*_*glob*_ and *E*_*loc*_ in 100 random control networks, respectively.

Modular organization is another feature of the brain network. A network can be fully subdivided into a set of non-overlapping modules *M* in a way that maximizes the number of within-module edges and minimizes the number of between-module edges. Then, modularity in the network can be defined as: 
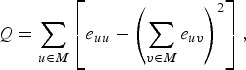
 where *e*_*uv*_ is the proportion of all edges that connect nodes in module *u* with nodes in module *v* (Blondel *et al.* 2008; Rubinov & Sporns, 2010). We used the Louvain algorithm (Blondel *et al.* 2008) to identify modular structure in the network which maximizes the modularity *Q*.

#### Local (nodal) network properties

To assess how much a central role each brain region (each node) takes part in the network, we estimated nodal degree, nodal efficiency and nodal betweenness centrality. Nodal degree *k*_*i*_ is defined as the number of edges linked to the node. A node with a high degree is more likely to have a central role in communication in the network, since it has many connections with other nodes in the network. Nodal efficiency is defined as average efficiency between the index node *i* and all other nodes in the network as the following: 
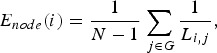
 where *L*_*i,j*_ is the shortest path length between each pair of nodes. Nodal betweenness centrality is a measure of the number of shortest paths mediated with the index node *i*. In other words, it represents the number of paths in the network that can be slowed or disconnected when the node *i* is removed. Nodal betweenness centrality is defined as: 
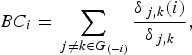
 where *δ*_*j,k*_ is the number of shortest paths between nodes *j* and *k* and *δ*_*j,k*_ is the number of shortest paths between nodes *j* and *k* that pass through node *i*.

Here we used global and local efficiency as a network metric reflecting small-world characteristics instead of using clustering coefficient and path length, because efficiency measures can be robustly estimated when some nodes are disconnected from the rest of the network (Latora & Marchiori, 2001).

In the present study, brain regions susceptible for MRI signal loss such as the orbitofrontal and medial temporal parts of the brain had disconnected nodes in some subjects even at edge density as high as 25%. However, there was no significant group difference in amplitude of BOLD rsfMRI fluctuation in these brain regions. We also confirmed our findings in global network properties with the network generated from the minimum spanning tree with additional edges above global threshold *τ* (Alexander-Bloch *et al.* 2010; Lin *et al.* 2013) in which all nodes were ensured to be connected.

### Statistical analysis for global and local network properties

Two-sample *t* tests were performed to assess group differences in clinical characteristics and graph-theoretical parameters between each patient group and the healthy control group using SPSS (version 17.0; USA). We used Pearson's χ^2^ test to estimate the difference in gender between groups. Significant between-group differences were determined at *p* < 0.05 (two-tailed). Bonferroni correction was used to control for multiple comparisons in testing regional network properties (*n* = 90 for the AAL atlas). Pearson's correlation *r* was examined for correlation between the brain network properties and individual covariates such as BMI and BES.

### NBS: region-to-region connectivity

For group comparisons in region-to-region connectivity, we utilized NBS (Zalesky *et al.* 2010), which deals with multiple comparisons by detecting clusters of connections that significantly differ across groups instead of testing individual connections. We used NBS to compare region-to-region connectivity *z*_*i,j*_ in the obese subjects and healthy controls using an initial threshold on the *t* statistics (*T* > 3) of individual edge differences as described in Zalesky *et al.* (2010). The interconnected graph component was identified in the set of the suprathreshold links with *t* statistic higher than a threshold of *T* = 3 in group comparison of region-to-region connectivity *z*_*i,j*_ (normalized correlation coefficient). A family-wise error (FWE)-corrected *p* value was computed for the size of the graph component (i.e. the number of interconnected links) using 10 000 permutation tests (*p* < 0.05, FWE-corrected).

## Results

### Demographic variables

There was no significant difference in gender ratio and age in the obese subjects with and without BED and healthy controls. BMI was significantly higher in both obese subgroups compared with healthy controls (*p* < 0.001, one-way analysis of variance; ANOVA), but did not differ between the two obese subgroups. There was also significant difference in BES across groups (*p* < 0.001, one-way ANOVA), with the obese BED patients exhibiting higher BES compared with the obese subjects without BED or healthy controls (Table 1). IQ and BDI were also significantly different between groups.

### Overall summary

**Fig. 1.**
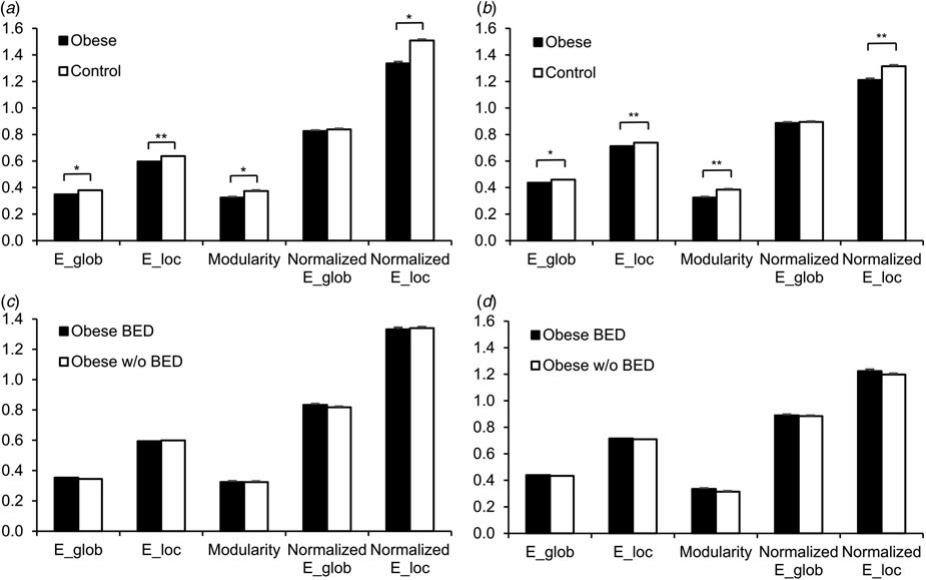
Alteration in global network properties in obese subjects. (*a* and *b*) Obese subjects (*n* = 40) showed reduced global efficiency (E_glob), local efficiency (E_loc), modularity and normalized local efficiency compared with healthy controls (*n* = 40). (*c* and *d*) Obese binge eating disorder (BED) patients (*n* = 20) and obese subjects without (w/o) BED (*n* = 20) did not differ in any global network properties (all *p* > 0.22). In (*a*) and (*c*), results are in the Automated Anatomical Labeling (AAL) atlas with 90 brain regions, and confirmed in (*b*) and (*d*) in the Harvard–Oxford (H-O) atlas with 470 equivalent parcellations. Values are means, with standard errors represented by vertical bars. * *p* < 0.01, ** *p* < 0.001.

**Fig. 2.**
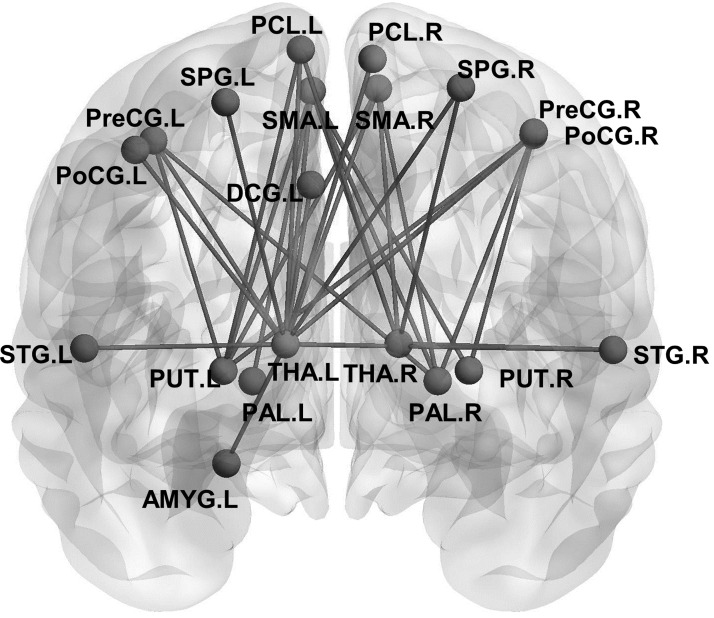
Decreased region-to-region functional connectivity in obese subjects. Comparison of region-to-region connectivity using network-based statistics controlling for multiple comparisons (*p* < 0.05, network-based statistics) (Automated Anatomical Labeling atlas with 90 brain regions; AAL90 atlas). This network represents decreased connectivity in all obese subjects compared with healthy controls. L, Left; R, right; PCL, paracentral lobule; SPG, superior parietal gyrus; PreCG, precentral gyrus; SMA, supplementary motor area; PoCG, postcentral gyrus; DCG, middle cingulate gyrus; STG, superior temporal gyrus; PUT, putamen; THA, thalamus; PAL, pallidum; AMYG, amygdala.

**Fig. 3.**
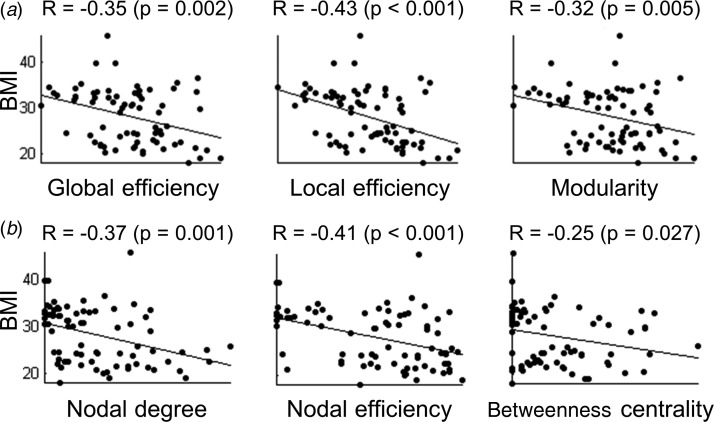
Correlation between network metrics and body mass index (BMI; kg/m^2^) across all subjects (*n* = 80). (*a*) Correlation between BMI and global network metrics across all subjects (Automated Anatomical Labeling atlas with 90 brain regions; AAL90 atlas). (*b*) Correlation between BMI and local network metrics focusing on the left putamen across all subjects (AAL90 atlas).

In the following we compared 40 obese *v*. 40 healthy controls along with comparisons of obese subjects with and without BED in: (i) global network metrics using AAL90 (Fig. 1*a, b*) with confirmation with H-O470 (Fig. 1*b, d*) and minimum spanning tree analysis; (ii) regional (nodal) network metrics using AAL90 (Table 2) and confirmation with H-O470 (online Supplementary Table S1); (iii) region-to-region connectivity (Fig. 2). BMI correlations with global (Fig. 3*a*), local (Fig. 3*b*) and network cluster connectivity weights are reported. Finally we also compare the result with single-echo rsfMRI analysis using conventional denoising (online Supplementary Table S2).

**Table 2.** Brain regions with abnormal nodal network characteristics in the entire group of obese subjects as compared with the healthy controls using the AAL90 atlas^*a*^

Network metric	Brain regions	Obese subjects	Healthy controls	*p*, Bonferroni corrected
Nodal degree				
Control > obese	Left thalamus	10.0	21.4	<0.001
	Right thalamus	11.0	20.4	0.002
	Right putamen	5.6	12.2	0.002
	Left putamen	4.7	10.2	0.007
	Right pallidum	1.4	4.2	0.042
Obese > control	Right superior occipital gyrus	19.5	15.4	0.037
Nodal efficiency				
Control > obese	Left thalamus	0.303	0.485	<0.001
	Right thalamus	0.310	0.477	0.003
	Left putamen	0.219	0.359	0.004
	Right pallidum	0.114	0.244	0.006
	Right putamen	0.234	0.376	0.008
	Right dorsal cingulate gyrus	0.547	0.588	0.014
	Right inferior frontal gyrus, opercular part	0.394	0.459	0.022
	Left pallidum	0.097	0.206	0.047
Nodal betweenness				
Control > obese	Left superior frontal gyrus	115	224	0.048

AAL, Automated Anatomical Labeling.

^a^ Reported in arbitrary units.

### Global network properties

We examined global network properties in the whole-brain networks constructed with parcellation using the AAL atlas and the H-O470 atlas as shown in Fig. 1. There were no group differences between the obese BED patients (*n* = 20) and the obese subjects without BED (*n* = 20) in any of global network metrics (all *p* > 0.22, Fig. 1*c, d*). Thus, we collapsed both obese groups with and without BED into one group of all obese subjects (*n* = 40) and compared this group with healthy controls (*n* = 40) in all subsequent data analyses. The global network properties in the obese BED patients subgroup (*n* = 20) *v*. healthy controls and the obese subgroup without BED (*n* = 20) *v*. healthy controls are reported in the online Supplementary material (see online Supplementary Fig. S1).

The obese subjects (*n* = 40) exhibited decreased global and local efficiency compared with healthy controls (*n* = 40) in the whole-brain network constructed with the 90 regions of the AAL atlas (*p* = 0.0012 and *p* = 0.0001, respectively; see Fig. 1*a*). We estimated normalized efficiency measures with respect to the randomly rewired control networks, and found that normalized local efficiency was significantly lower in the obese subjects compared with healthy controls in both atlases (*p* = 0.001). In addition, modularity in the whole-brain network was also significantly reduced in the obese group compared with healthy controls (*p* = 0.002). Taken together, these findings suggest decreased small-world characteristics and poor modularization of the network in the obese subjects. In contrast, there was no group difference in normalized global efficiency, and the observed difference in global efficiency might be attributed to group differences in degree distribution (see online Supplementary Fig. S2). The obese subjects had a larger number of nodes with few connections (*k* ⩽ 1) which are more likely to be distal or isolated in the network.

All these group differences in global network properties were observed in the whole-brain network consisting of 470 regions in the H-O470 atlas as well (Fig. 1*b*). We also found group differences in global efficiency, local efficiency, modularity and normalized local efficiency within the whole-brain network constructed from the minimum spanning tree with additional edges with global thresholding (all *p* < 0.05, data not shown).

### Regional network properties

We then identified brain regions with a significant difference in regional (nodal) network properties between the obese group and their matched healthy controls. Using the AAL atlas, we found a significant decrease in nodal degree and nodal efficiency in the bilateral putamen, thalamus and right pallidum (*p* < 0.05, Bonferroni correction; Table 2), reflecting a decreased number of connections and reduced efficiency of information flow with these subcortical regions. Using the H-O470 atlas, we found a similar decrease in nodal degree and efficiency in subparcellated regions of the putamen, thalamus and pallidum (*p* < 0.05, Bonferroni correction; see online Supplementary Table S1). Although other regions also showed differences between groups, we focus here only on the regions replicated across both atlases. No significant difference was found between the obese BED patients and the obese subjects without BED at the same significance level (all *p* > 0.37, Bonferroni correction).

### NBS

Using NBS, we compared the region-to-region functional connectivity measure, normalized correlation coefficients across the regional mean rsfMRI signals in the AAL atlas, between the obese group and healthy controls. NBS identified a network cluster of significantly decreased functional connectivity in the cortico-striatal/cortico-thalamic network in the obese groups compared with healthy controls (*p* < 0.05, FWE-corrected; see Fig. 2). The network cluster of decreased functional connectivity in the obese subjects consisted of the bilateral putamen, pallidum and thalamus as well as cortical regions associated with motor/somatosensory and associative function such as the primary motor cortex (precentral gyrus), SMA, paracentral lobule, primary somatosensory gyrus (postcentral gyrus), superior parietal lobule and superior temporal cortex. In addition, the left amygdala was also included in this cluster of decreased connectivity. NBS did not identify a cluster of significantly increased functional connectivity in the obese subjects compared with healthy controls. With the H-O470 atlas, we did not expect to find and did not find significant differences using NBS analysis, as parcellation using this atlas is associated with a markedly larger number of multiple comparisons (470 × 469/2) relative to the degree of freedom (i.e. number of volumes, *n* = 239) in our rsfMRI data.

### Correlation with clinical variables

We estimated correlations between clinically relevant measures such as BMI and BES and global network metrics using Pearson correlations. BMI in all subjects (*n* = 80) was negatively correlated with global efficiency, local efficiency, modularity and normalized local efficiency (all *R* < −0.32 and *p* < 0.01; see Fig. 3*a*), suggesting a less efficient and less modular organization of the brain network in subjects with higher BMI. These correlations remained significant after regressing out the effect of age. No significant correlation was found with BES, IQ or BDI scores across individuals (all *p* > 0.20).

We also examined correlations with clinical measures and regional network metrics of regions significantly implicated in the regional network analyses, specifically, the bilateral putamen, pallidum and thalamus. BMI was negatively correlated with nodal degree and efficiency in these six subcortical regions (all *R* < −0.27 and *p* < 0.05; see Fig. 3*b* for left putamen results) as well as nodal betweenness centrality of left thalamus and left putamen (*R* = −0.34 and *p* = 0.003, and *R* = −0.25 and *p* = 0.027, respectively). BES was negatively correlated with nodal degree and efficiency in the bilateral putamen (all *R* < −0.24 and *p* < 0.05), but these correlations with BES were not preserved after controlling the effect of BMI.

Average connectivity weights in the network cluster identified with NBS were also negatively correlated with BMI and BES (*R* = −0.30 and *p* = 0.008, and *R* = −0.27 and *p* = 0.026, respectively). However, correlation with BES did not remain significant after controlling the effect of BMI.

### Comparison with the conventional single-echo analysis

To compare these findings with conventional rsfMRI analyses, we conducted the same graph-theoretical analysis but using a conventional single-echo fMRI denoising method (regressing out head motion and bandpass filtering) instead of ME-ICA. In this single-echo fMRI analysis, the obese subjects did not show any difference from their matched healthy control group in global network characteristics except decreased local efficiency at a trend level (*p* = 0.065; see online Supplementary Table S2). No alteration in nodal network properties in the thalamic and striatal regions was found in the single-echo fMRI analysis.

## Discussion

We compared whole-brain network properties of obese subjects using data-driven graph-theoretical approaches and highlighted convergent findings across two atlases differing by regions and number of parcellations. The obese subjects exhibited alterations in global network properties, particularly decreased local efficiency and modularity. Reduced modularity suggested disrupted modular organization of the network and poor functional segregation as well. In comparison with random control networks, normalized local efficiency was specifically impaired, indicating that local clustering structures were disrupted, becoming closer to a random network compared with healthy controls. Taken together with the lack of group difference in normalized global efficiency, these findings correspond to reduced small-world characteristics in the brain network of obese subjects. The normalized global efficiency in both control and obese groups was of a similar level comparable with random networks; thus any further random-like organization in the obese subjects did not further affect global efficiency in the brain network. Global efficiency was rather decreased in the obese subjects due to their degree distribution containing a larger number of distal or isolated nodes of degree *k* ⩽ 1. Clinically relevant measures of BMI were also negatively correlated with all of the implicated global network metrics.

There are a limited number of studies applying graph-theoretical analysis with density-based thresholding in rsfMRI data in substance abuse (Breckel *et al.* 2013; Jiang *et al.* 2013; Lin *et al.* 2015; Sjoerds *et al.* 2015; Wang *et al.* 2015). A previous study in heroin-dependent individuals (Jiang *et al.* 2013) found a significantly lower normalized clustering coefficient and small-world characteristics which are in accordance with our findings of decreased normalized local efficiency. Similarly, decreased local efficiency and small-world characteristics were reported in chronic substance abusers (Wang *et al.* 2015). Our finding suggests that a network disruption in local clustering structure and small-world characteristics might link substance abuse and pathological misuse of food.

Beyond these global network alterations, the regional (nodal) network properties and region-to-region connectivity revealed alterations mainly in subcortical regions including the bilateral thalamus, putamen and pallidum using a data-driven hypothesis-free analysis. Profound alterations in nodal degree and efficiency were found in the putamen, pallidum and thalamus in the obese subjects consistently across two separate parcellation atlases after a stringent Bonferroni correction for multiple comparison. Decreased nodal degree and efficiency indicated a reduced number of connections and communication efficiency in the putamen, pallidum and thalamus with other brain regions. Convergent with decreased nodal degree in the striatal and thalamic regions, the obese group also exhibited decreased functional connectivity in a cortico-striatal/cortico-thalamic network involving the bilateral putamen, pallidum and thalamus with cortical regions that encompass motor and associative function in addition to the left amygdala. Correlation with BMI and BES scores suggested that these regional alterations are particularly associated with obesity and maladaptive eating behaviour.

Although our findings were data-driven and hypothesis-free, the observations dovetail with theories of habit formation in which positive reinforcement through long-term drug exposure shifts flexible goal-directed behaviours towards automatic inflexible habitual behaviours implicating the putamen (Everitt *et al.* 2008). These findings in the putamen build on two smaller studies demonstrating impairments in putaminal connectivity in obesity and correlating with insulin sensitivity but demonstrating an increase rather than a decrease in connectivity using independent components analysis of resting-state data (Kullmann *et al.* 2012; Garcia-Garcia *et al.* 2013). Converging evidence implicates a role for lower D_2/3_ receptors in the striatum and particularly the dorsolateral striatum (putamen) in obesity and binge eating in rodent and human studies (Wang *et al.* 2001; de Weijer *et al.* 2011). Rodent binge eating models suggest a role for lower D_2_ receptor binding particularly in the dorsolateral striatum (Colantuoni *et al.* 2001; Bello *et al.* 2002). Whether the D_2_ receptor levels are predictive of or secondary to obesity remains to be established. Disrupted nodal degree and clustering coefficient in the left caudate and bilateral putamen have also been associated with alcohol dependence severity and duration (Sjoerds *et al.* 2015).

We further showed that the ME-ICA rsfMRI is associated with significant group differences in global network metrics compared with single-echo rsfMRI using conventional denoising techniques, which is consistent with reports of an enhanced signal:noise ratio in ME-ICA (Kundu *et al.* 2012, 2013). It emphasizes the relevance of more sensitive techniques to demonstrate group differences of the resting-state brain network in clinical studies. In the present study, the smaller sample size of obese BED patients and obese subjects without BED subgroups might limit any differences in network alteration associated with BED.

Using graph-theoretical analysis, we revealed altered network topological structures in obesity in both whole-brain network and regional levels. A novel ME-ICA technique in the present study enabled detection of group differences with a stringent statistical threshold. We emphasize global impairments in network efficiency in obesity with disrupted local network organization closer to random networks. We further highlight impairments in cortico-striatal/cortico-thalamic circuitry focusing on putaminal and cortical motor regions consistent with abnormalities in striatal dopaminergic processing in obesity. The network alterations found in the present study were primarily associated with severity of obesity (i.e. BMI); thus one might need to take into account obesity as a potential confounding factor in group analysis of brain network properties particularly in graph theory analysis. Our findings highlight the role of network properties in pathological food misuse as possible biomarkers and therapeutic targets.

## Supplementary material

For supplementary material accompanying this paper visit http://dx.doi.org/10.1017/S0033291716002646. click here to view supplementary material
