# Supplementary material for: Disrupted resting-state brain network properties in obesity: decreased global and putaminal cortico-striatal network efficiency
Source: Psychol Med. 2016 Nov 2;47(4):585–96. doi: 10.1017/S0033291716002646 (PMC5426347; doi:10.1017/S0033291716002646)
Supplement: Supplementary file 1 [file S0033291716002646sup001.doc]

**Supplementary material**

**Supplementary Table S1.** *Brain regions with abnormal nodal network characteristics in the entire group of obese subjects as compared with the healthy controls (HO470 atlas)*

Reported in arbitrary units.

| **Network metric** | **Brain regions** | **Obese subjects** | **Healthy controls** | **p-value,**  **Bonferroni corrected** |
| --- | --- | --- | --- | --- |
| **Nodal degree** |  |  |  |  |
| HV > Obese | R. Thalamus (02) | 44.2 | 110.6 | <.001 |
|  | L. Thalamus (02) | 39.9 | 98.7 | <.001 |
|  | L. Putamen (01) | 26.4 | 62.8 | .001 |
|  | R. Putamen (01) | 23.3 | 62.0 | .001 |
|  | L. Thalamus (01) | 52.8 | 107.6 | .001 |
|  | R. Putamen (03) | 20.5 | 46.1 | .020 |
|  | R. Frontal operculum cortex (10) | 47.1 | 80.2 | .024 |
|  | R. Thalamus (01) | 63.0 | 112.0 | .029 |
|  | L. Thalamus (04) | 46.9 | 94.0 | .033 |
|  | L. Putamen (03) | 17.7 | 40.1 | .041 |
| Obese > HV | L. Superior parietal lobule (10) | 102.3 | 63.3 | .005 |
| **Nodal efficiency** |  |  |  |  |
| HV > Obese | R. Thalamus (02) | 0.400 | 0.549 | <.001 |
|  | L. Thalamus (02) | 0.398 | 0.532 | <.001 |
|  | R. Pallidum (01) | 0.124 | 0.299 | .001 |
|  | L. Thalamus (01) | 0.427 | 0.546 | .002 |
|  | L. Thalamus (04) | 0.399 | 0.529 | .003 |
|  | R. Putamen (01) | 0.306 | 0.452 | .007 |
|  | R. Thalamus (01) | 0.441 | 0.551 | ..017 |
|  | L. Putamen (01) | 0.339 | 0.461 | .018 |
|  | R. Thalamus (04) | 0.418 | 0.534 | .020 |
|  | R. Inferior frontal gyrus,  pars opercularis (01) | 0.416 | 0.501 | .030 |
|  | R. Frontal operculum cortex (01) | 0.414 | 0.507 | .040 |
| **Nodal betweeness centrality** |  |  |  |  |
| HV > Obese | R. Thalamus (02) | 547 | 1476 | .005 |

**Supplementary Table S2.** *Group difference in resting state brain network properties using conventional single-echo denoised data*

(A) Global network properties

| Network metrics,  Mean (SD) | Global efficiency | Local efficiency | Modularity | Normalized global eff. | Normalized local eff. |
| --- | --- | --- | --- | --- | --- |
| Obese subjects (N = 40) | .373 (.042) | .621 (.044) | .348 (.067) | .806 (.051) | 1.438 (.306) |
| Control (N = 40) | .387 (.035) | .639 (.042) | .371 (.059) | .820 (.043) | 1.517 (.262) |
| p-value | .104 | .065 | .102 | .176 | .217 |

(B) Local network properties: Only nodal efficiency in the right putamen remained significant at the statistical threshold of p = 0.05, Bonferroni corrected.

| Network metric | Brain regions | Obese subjects (N = 40) | Healthy controls (N = 40) | p-value  (Bonferroni corrected) |
| --- | --- | --- | --- | --- |
| Nodal efficiency |  |  |  |  |
| Control > Obese | R. Putamen | 0.537 | 0.727 | .008 |

**Supplementary Fig. S1.** Comparison of global network properties between the two types of obese subgroups vs. healthy controls (HV). (A~B) Obese BED patients subgroup (N = 20) showed reduced global efficiency, local efficiency, modularity and normalized local efficiency compared to healthy controls (N = 40). (C~D) Obese subgroup without BED (N = 20) showed reduced global efficiency, local efficiency, modularity and normalized local efficiency compared to healthy controls (N = 40). (A, C) are results in the AAL atlas with 90 brain regions, and (B, D) are in the H-O470 atlas. Mean ± S.E.M. *: p < 0.05. **: p < 0.01. ***: p < 0.001

**
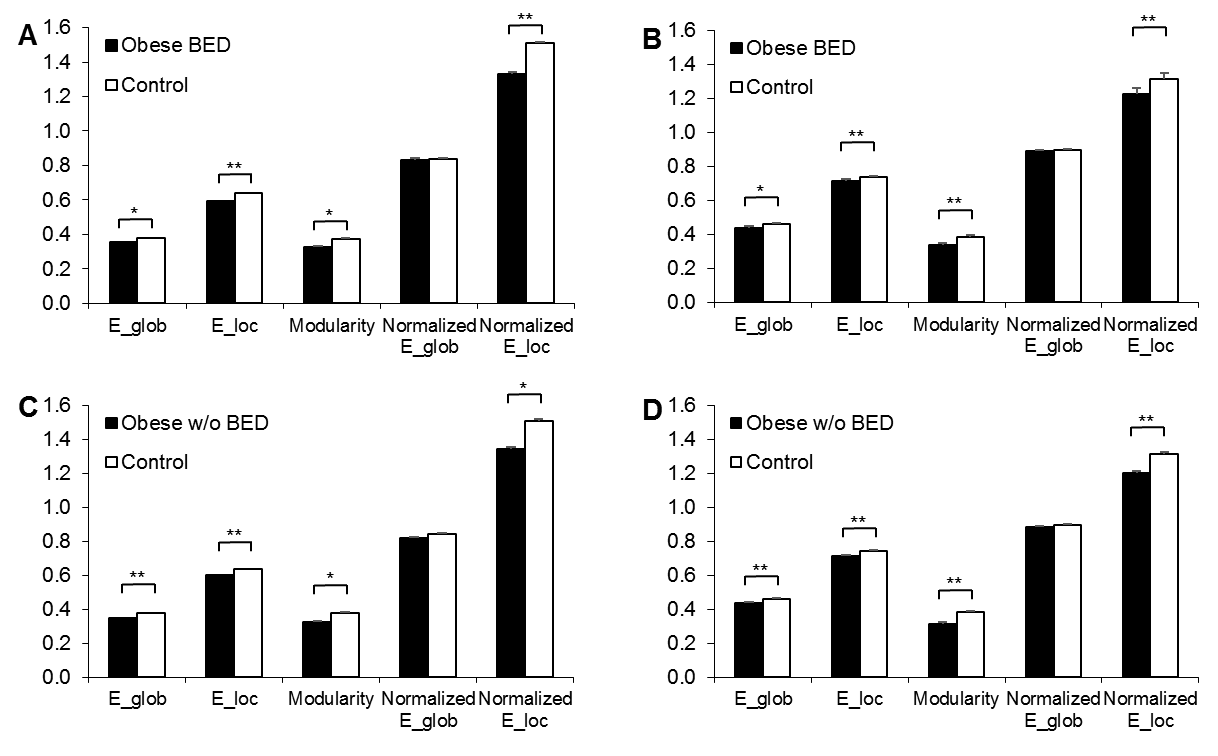
**

**Supplementary Fig. S2.** Group difference in the degree distribution (AAL90 atlas). (Left) The obese group (red) had significantly larger number of nodes with few connections (degree k ≤ 1) which are likely to be distal or isolated in the network. The obese group had more nodes with high degree (k ~ 20, 26) but the size of the difference was small. In contrast, the healthy controls (black) tended to have more nodes with a middle range of degree (k ~ 9, 16, 17). (Right) The obese group showed a lower slope in the cumulative degree distribution, i.e. more nodes with extremely low degree or extremely high degree.

**Degree distribution (original) Degree distribution (cumulative)**

**
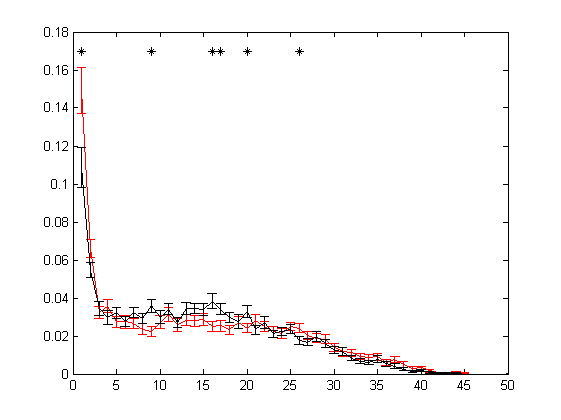

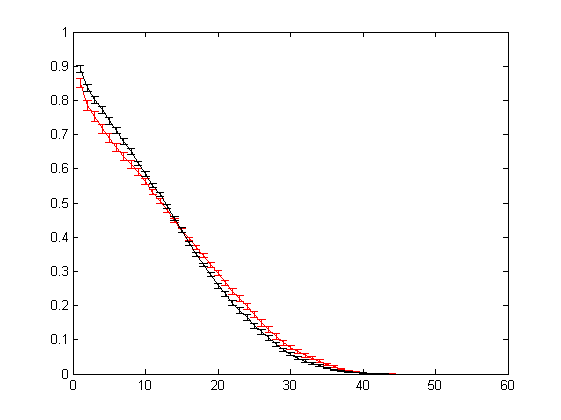
**
